# Trends in Racial and Ethnic Diversity in Internal Medicine Subspecialty Fellowships From 2006 to 2018

**DOI:** 10.1001/jamanetworkopen.2019.20482

**Published:** 2020-02-07

**Authors:** Lekshmi Santhosh, Jennifer M. Babik

**Affiliations:** 1 Division of Pulmonary and Critical Care Medicine, Department of Medicine, University of California, San Francisco; 2 Division of Infectious Diseases, Department of Medicine, University of California, San Francisco

## Abstract

This cross-sectional study examines trends in racial/ethnic diversity in internal medicine subspecialty fellowships between 2006 and 2018.

## Introduction

Workforce diversity has been increasingly emphasized in academic medicine, especially regarding recruitment and retention of physicians underrepresented in medicine (UIM).^1^ In 2019, Stone et al^2^ examined gender in internal medicine (IM) subspecialty fellowships and showed substantial differences between subspecialties. However, the racial and ethnic diversity of IM residents and subspecialty fellows has not been described, to our knowledge.

## Methods

We performed a cross-sectional study of race/ethnicity in IM residents and subspecialty fellows using published Graduate Medical Education census reports from 2006, when race/ethnicity data were first included, to 2018 (eAppendix in the Supplement). The University of California, San Francisco, institutional review board does not require approval for research using publicly available, deidentified data. This report follows Strengthening the Reporting of Observational Studies in Epidemiology (STROBE) reporting guideline for cross-sectional studies.

We manually extracted and validated data for IM residents and fellows in the 9 following IM subspecialties: cardiology, endocrinology, gastroenterology, geriatrics, infectious diseases, hematology/oncology, nephrology, pulmonary/critical care medicine, and rheumatology. Allergy/immunology and hospice/palliative medicine were excluded because they are not restricted to IM trainees.

The Association of American Medical Colleges defines UIM as “racial and ethnic populations that are underrepresented in the medical profession relative to their numbers in the general population,”^3^ and the Association of American Medical Colleges Medical Minority Applicant Registry further describes UIM as individuals who self-identify as African American or black, Hispanic or Latino, American Indian or Alaska Native, or Native Hawaiian or Pacific Islander.^4^ Trainees whose race/ethnicity was reported as multiracial or other/unknown were not considered UIM. We obtained US population data for 2018 from the US Census Bureau.^5^

To determine whether there was a significant change in proportions during the study period, we performed the χ^2^ test for trend (Cochran-Armitage test) using Prism version 8.3.0 (GraphPad Software); results were considered statistically significant at a 2-tailed *P* < .05.

## Results

A total of 298 820 IM residents and 124 382 subspecialty fellows were included in our sample; 2184 residents (0.7%) and 1001 fellows (0.8%) who identified as multiracial and 42 377 residents (14.2%) and 8222 fellows (6.6%) whose race/ethnicity was other or unknown were excluded. Between 2006 and 2018, the annual number of IM residents and subspecialty fellows increased (from 21 855 to 26 228 residents and 8144 to 10 578 fellows) (Table). Over time, the percentage of UIM trainees was unchanged in IM residencies (2688 [12.3%; 95% CI, 11.9%-12.7%] to 3599 [13.7%; 95% CI, 13.3%-14.1%]; *P* = .28) but increased in all subspecialty fellowships (874 [10.7%; 95% CI, 10.1%-11.4%] to 1299 [12.3%; 95% CI, 11.7%-12.9%]; *P* < .001) (Table; Figure, A). However, there were substantial differences by subspecialty (Table; Figure, B). The percentage of UIM fellows was unchanged in geriatrics (63 of 301 [20.9%; 95% CI, 16.7%-25.9%] to 31 of 214 [14.5%; 95% CI, 10.4%-19.8%; *P* = .19), hematology/oncology (84 of 1164 [7.2%; 95% CI, 5.9%-8.8%] to 154 of 1674 [9.2%; 95% CI, 7.9%-10.7%]; *P* = .28), pulmonary/critical care medicine (114 of 1087 [10.5%; 95% CI, 8.8%-12.4%] to 178 of 1726 [10.3%; 95% CI, 9.0%-11.8%]; *P* = .31), and rheumatology (42 of 366 [11.5%; 95% CI, 8.6%-15.1%] to 57 of 477 [11.9%; 95% CI, 9.4%-15.2%]; *P* = .90) but increased in cardiology (206 of 2142 [9.6%; 95% CI, 8.4%-10.9%] to 316 of 2731 [11.6%; 95% CI, 10.4%-12.8%]; *P* < .001), endocrinology (56 of 486 [11.5%; 95% CI, 9.0%-14.7%] to 115 of 666 [17.3%; 95% CI, 14.6%-20.3%]; *P* < .001), gastroenterology (101 of 1097 [9.2%; 95% CI, 7.6%-11.1%] to 184 of 1525 [12.1%; 95% CI, 10.5%-13.8%]; *P* < .001), infectious diseases (98 of 679 [14.4%; 95% CI, 12.0%-17.3%] to 133 of 731 [18.2%; 95% CI, 15.6%-21.2%]; *P* < .001), and nephrology (110 of 822 [13.4%; 95% CI, 11.2%-15.9%] to 131 of 834 [15.7%; 95% CI, 13.4%-18.3%]; *P* < .001). The highest percentage of UIM fellows over time was in infectious diseases, and the lowest was in hematology/oncology.

**Table.** Total and UIM IM Residents and Subspecialty Fellows in 2006 and 2018

Specialty	2006		2018		*P* Value^a^
	Total, No.	UIM, No. (%) [95% CI, %]	Total, No.	UIM, No. (%) [95% CI, %]	
IM residents	21 885	2688 (12.3) [11.9-12.7]	26 228	3599 (13.7) [13.3-14.1]	.28
All subspecialty fellows	8144	874 (10.7) [10.1-11.4]	10 578	1299 (12.3) [11.7-12.9]	<.001
Cardiology	2142	206 (9.6) [8.4-10.9]	2731	316 (11.6) [10.4-12.8]	<.001
Endocrinology	486	56 (11.5) [9.0-14.7]	666	115 (17.3) [14.6-20.3]	<.001
Gastroenterology	1097	101 (9.2) [7.6-11.1]	1525	184 (12.1) [10.5-13.8]	<.001
Geriatrics	301	63 (20.9) [16.7-25.9]	214	31 (14.5) [10.4-19.8]	.19
Hematology/oncology	1164	84 (7.2) [5.9-8.8]	1674	154 (9.2) [7.9-10.7]	.28
Infectious diseases	679	98 (14.4) [12.0-17.3]	731	133 (18.2) [15.6-21.2]	<.001
Nephrology	822	110 (13.4) [11.2-15.9]	834	131 (15.7) [13.4-18.3]	<.001
PCCM	1087	114 (10.5) [8.8-12.4]	1726	178 (10.3) [9.0-11.8]	.31
Rheumatology	366	42 (11.5) [8.6-15.1]	477	57 (11.9) [9.4-15.2]	.90

Abbreviations: IM, internal medicine; PCCM, pulmonary/critical care medicine; UIM, underrepresented in medicine.

^a^ *P* value is for the χ^2^ test for trend (Cochran-Armitage test) for data from all years between 2006 and 2018.

**Figure.**
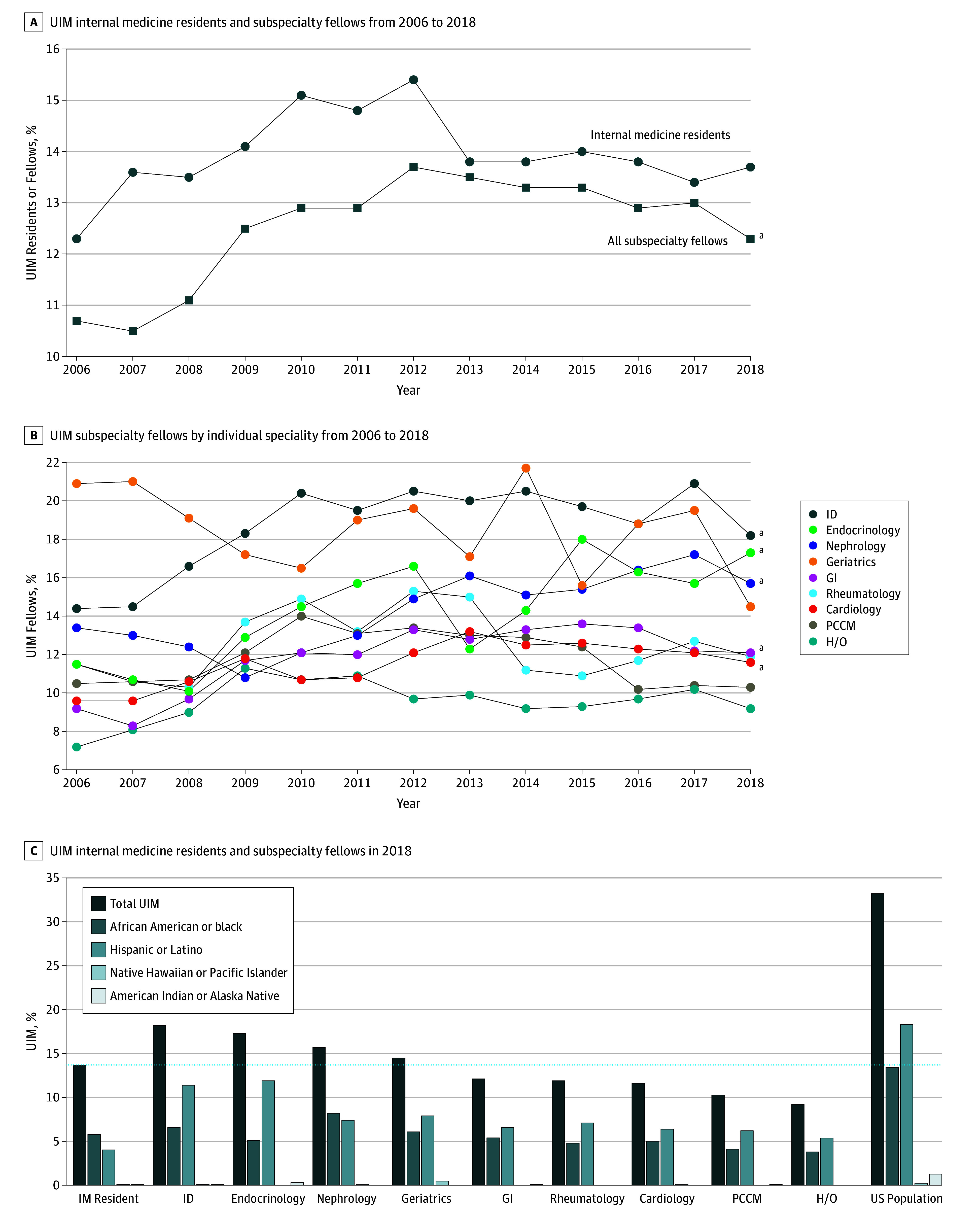
Percentage of Underrepresented in Medicine (UIM) Internal Medicine Residents and Subspecialty Fellows, 2006 to 2018 A, The percentage of UIM trainees for internal medicine residents and subspecialty fellows, with all subspecialties combined. B, The percentage of UIM subspecialty fellows by subspecialty. C, The percentage of UIM trainees in internal medicine residencies and subspecialty fellowships in 2018. The horizontal line indicates the percentage of UIM trainees in internal medicine residencies. H/O indicates hematology/oncology; ID, infectious diseases; GI, gastroenterology; and PCCM, pulmonary/critical care medicine. ^a^*P* < .001.

The data on UIM residents and fellows from 2018 are shown in the Table and Figure, C. Compared with IM residencies, there were 4 subspecialty fellowships with higher percentages of UIM trainees (ie, infectious diseases, endocrinology, nephrology, and geriatrics) and 5 with lower percentages (ie, gastroenterology, rheumatology, cardiology, pulmonary/critical care medicine, and hematology/oncology). All specialties had considerably lower percentages of UIM trainees than in the US population (US population: black or African American, 14%; American Indian or Alaska Native, 1.3%; Native Hawaiian or other Pacific Islander, 0.2%; Hispanic or Latino, 18.3%).^5^

## Discussion

Between 2006 and 2018, the overall percentage of UIM subspecialty fellows increased but with substantial variation by specialty. The current state of diversity in subspecialty fellowships still requires attention. In 2018, no specialties reflected the diversity of the US population, and more than half had lower percentages of UIM trainees than IM residencies—a useful benchmark, given that residents are the direct pipeline for subspecialty fellows. With the exception of rheumatology, the subspecialties with the lowest percentages of UIM fellows were also the largest fellowships and the more procedural specialties. This raises questions about possible factors that may be affecting career choice for UIM residents; further research is needed to elucidate these. Moreover, there is overlap between the UIM and gender breakdown in IM subspecialties,^2^ with higher percentages of UIM and women in specialties such as infectious diseases, endocrinology, and geriatrics.

A limitation of our study is that Graduate Medical Education census data are imported from Association of American Medical Colleges databases with self-designated race/ethnicity when available, but otherwise, race/ethnicity is reported by program directors. Because fellows are the future subspecialty workforce, there is an urgent need to recruit and retain UIM trainees in all IM subspecialties, in particular in those with a disproportionately low and/or unchanged percentage of UIM trainees over time.

## Notes

Supplement. **eAppendix.** List of Data Sources

## References

[1] Lett LA, Murdock HM, Orji WU, Aysola J, Sebro R. Trends in racial/ethnic representation among US medical students. JAMA Netw Open. 2019;2(9):e1910490-e1910490. doi:10.1001/jamanetworkopen.2019.10490 31483469 PMC6727686

[2] Stone AT, Carlson KM, Douglas PS, Morris KL, Walsh MN. Assessment of subspecialty choices of men and women in internal medicine from 1991 to 2016 [published online September 23, 2019]. JAMA Intern Med. doi:10.1001/jamainternmed.2019.3833 31545349 PMC6763985

[3] Association of American Medical Colleges. Underrepresented in medicine definition. https://www.aamc.org/what-we-do/mission-areas/diversity-inclusion/underrepresented-in-medicine. Accessed November 27, 2019.

[4] Association of American Medical Colleges. Medical Minority Applicant Registry (MedMAR). https://students-residents.aamc.org/choosing-medical-career/article/medical-minority-applicant-registry-med-mar/. Accessed November 27, 2019.

[5] US Census Bureau. QuickFacts. https://www.census.gov/quickfacts/fact/table/US/SEX255218. Accessed August 9, 2019.

