# Error in Results Section

**DOI:** 10.1001/jamanetworkopen.2020.4401

**Published:** 2020-03-31

In the Research Letter titled “Trends in Racial and Ethnic Diversity in Internal Medicine Subspecialty Fellowships From 2006 to 2018,”^1^ published February 7, 2020, there was an error in the percentage of the black or African American US population listed in the last sentence of the Results section. It should have appeared as 14%. This article has been corrected.^1^
