# Supplementary material for: Trends in Racial and Ethnic Diversity in Internal Medicine Subspecialty Fellowships From 2006 to 2018
Source: JAMA Netw Open. 2020 Feb 7;3(2):e1920482. doi: 10.1001/jamanetworkopen.2019.20482 (PMC12543390; doi:10.1001/jamanetworkopen.2019.20482)
Supplement: Supplement. — eAppendix. List of Data Sources [file jamanetwopen-e1920482-s001.pdf]

Supplementary Online Content

Santhosh L, Babik JM. Trends in racial and ethnic diversity in internal medicine subspecialty fellowships from 2006 to 2018. *JAMA Netw Open*. 2020;3(2):e1920482. doi:10.1001/jamanetworkopen.2019.20482

eAppendix. List of Data Sources

This supplementary material has been provided by the authors to give readers additional information about their work.

© 2020 Santhosh L et al. *JAMA Network Open.*

eAppendix. List of Data Sources.

Brotherton SE, Etzel SI. Graduate Medical Education, 2006-2007. *JAMA*. 2007;298(9):1081-1096.

Brotherton SE, Etzel SI. Graduate Medical Education, 2007-2008. *JAMA*. 2008;300(10):1228.

Brotherton SE, Etzel SI. Graduate medical education, 2008-2009. *JAMA*. 2009;302(12):1357-1372.

Brotherton SE, Etzel SI. Graduate medical education, 2009-2010. *JAMA*. 2010;304(11):1255-1270.

Brotherton SE, Etzel SI. Graduate medical education, 2010-2011. *JAMA*. 2011;306(9):1015-1030.

Brotherton SE, Etzel SI. Graduate medical education, 2011-2012. *JAMA*. 2012;308(21):2264-2279.

Brotherton SE, Etzel SI. Graduate medical education, 2012-2013. *JAMA*. 2013;310(21):2328-2346.

Brotherton SE, Etzel SI. Graduate medical education, 2013-2014. *JAMA*. 2014;312(22):2427-2445.

Brotherton SE, Etzel SI. Graduate medical education, 2014-2015. *JAMA*. 2015;314(22):2436-2454.

Brotherton SE, Etzel SI. Graduate medical education, 2015-2016. *JAMA*. 2016;316(21):2291-2310.

Brotherton SE, Etzel SI. Graduate medical education, 2016-2017. *JAMA*. 2017;318(23):2368-2387.

Brotherton SE, Etzel SI. Graduate medical education, 2017-2018. *JAMA*. 2018;320(10):1051-1070.

© 2020 Santhosh L et al. *JAMA Network Open.*
